# Evaluation of a long-lasting microbial larvicide against *Culex quinquefasciatus* and *Aedes aegypti* under laboratory and a semi-field trial

**DOI:** 10.1186/s13071-024-06465-5

**Published:** 2024-09-14

**Authors:** Hyago Luiz Rique, Heverly Suzany Gouveia Menezes, Maria Alice Varjal Melo-Santos, Maria Helena Neves Lobo Silva-Filha

**Affiliations:** Departament of Entomology, Instituto Aggeu Magalhães-Fiocruz, Av. Moraes Rego S/N, Recife, PE 50740-465 Brazil

**Keywords:** *Lysinibacillus sphaericus*, Bti, Vectomax, Mosquito control, Resistance, Bacterial toxins

## Abstract

**Background:**

Microbial larvicides containing both *LysiniBacillus sphaericus* and *Bacillus thuringiensis* svar. *israelensis* (Bti) insecticidal crystals can display advantages for mosquito control. This includes a broader action against larvae that are refractory to the Binary (Bin) toxin from *L. sphaericus*, as Bin-resistant *Culex quinquefasciatus* and *Aedes aegypti* naturally refractory larvae, which often co-habit urban areas of endemic countries for arboviruses. Our principal goal was to assess the toxicity of a combined *L. sphaericus*/Bti larvicide (Vectomax FG™) to *Cx. quinquefasciatus* (susceptible CqS and Bin-resistant CqR) and *Ae. aegypti* (Rocke) and to determine its persistence in the breeding sites with those larvae.

**Methods:**

The toxicity of a combined *L. sphaericus*/Bti product (VectoMax FG™) to larvae was performed using bioassays, and persistence was evaluated in simulate field trials carried out under the shade, testing two label concentrations during 12 weeks. A laboratory strain SREC, established with CqS and CqR larvae, was kept during four generations to evaluate the ability of the *L. sphaericus*/Bti to eliminate resistant larvae.

**Results:**

The *L. sphaericus*/Bti showed toxicity (mg/L) to larvae from all strains with a decreasing pattern for CqS (LC_50_ = 0.006, LC_90_ = 0.030), CqR (LC_50_ = 0.009, LC_90_ = 0.069), and Rocke (LC_50_ = 0.042, LC_90_ = 0.086). In a simulated field trial, the larvicide showed a persistence of 6 weeks and 8 weeks, controlling larvae from all strains in containers with 100 L of water, using 2 g or 4 g per container (100 L), respectively. The treatment of SREC larvae with *L. sphaericus*/Bti showed its capacity to eliminate the Bin-resistant individuals using suitable concentrations to target those larvae.

**Conclusions:**

Our results showed the high efficacy and persistence of the *L*. *sphaericus*/Bti larvicide to control *Cx. quinquefasciatus* and *Ae. aegypti* that might cohabit breeding sites. These findings demonstrated that such larvicides can be an effective tool for controlling those species in urban areas with a low potential for selecting resistance.

**Graphical Abstract:**

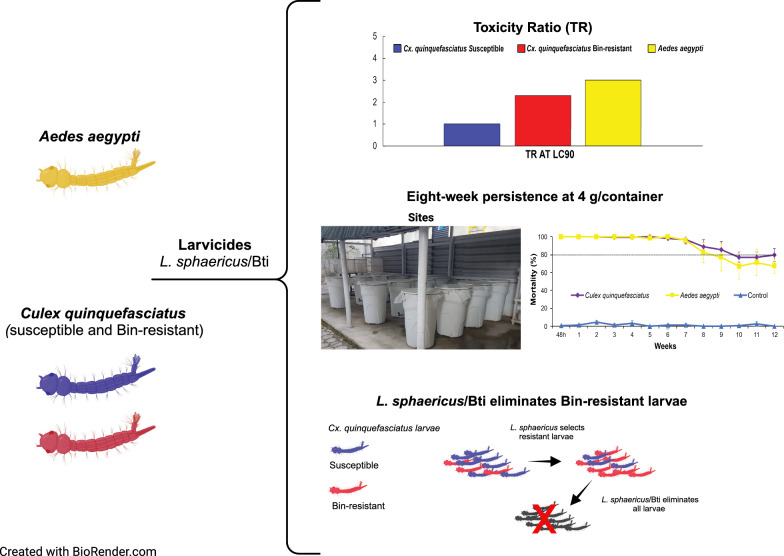

**Supplementary Information:**

The online version contains supplementary material available at 10.1186/s13071-024-06465-5.

## Background

The microbial larvicides based on *LysiniBacillus sphaericus* or *Bacillus thuringiensis* svar. *israelensis* (Bti) can be used to control mosquito species of medical importance within integrated programs [1], and recently, products based on both bacteria have been developed. The active ingredient produced by those bacteria are insecticidal crystals with protoxins that, upon ingestion by mosquito larvae, are activated into toxins and bind to specific receptors on the midgut epithelial cells [2, 3]. The insecticidal *L. sphaericus* crystals have a single protoxin called binary (Bin), that have been mostly used for controlling *Culex* and *Anopheles* species. The Bin toxin action on the major target species, *Culex quinquefasciatus* and *Culex pipiens*, depends on its binding to specific receptors that are the α-glucosidases, named Cqm1/Cpm1, which are attached to the midgut epithelial cells by a glycosylphosphatidylinositol (GPI) anchor, as reviewed by Silva-Filha et al. [4]. The action of the Bin depends on this membrane-bound receptor, and resistance often is provoked by the lack of those receptors in the midgut [5, 6]. *Ae. aegypti* larvae are naturally refractory to the Bin toxin due to the absence of such specific midgut receptors [5, 7]. The Bti insecticidal crystal contains four major protoxins (Cry11Aa, Cry4Ba, Cry4Aa, and Cyt1Aa) that act in synergy and are toxic to Culicidae, as well as Simuliidae, larvae [8, 9]. The Cyt1Aa plays a central role for the synergy of these toxins because it promotes the binding of the Cry toxins with high affinity to receptors in the epithelium [10, 11]. The Cry toxins from Bti can specifically bind to different receptors, which are GPI-anchored proteins in the midgut of larvae such as aminopeptidases, cadherins, alkaline phosphatases, and α-amylases [12], and these interactions can also be modulated by other molecules such as lectins [13]

*L. sphaericus*- and Bti-based larvicides are effective for mosquito control, but they display some limitations. For *L. sphaericus*, a major issue can be the resistance to the Bin toxin already reported for *Cx. quinquefasciatus* or *Cx. pipiens* field-treated populations [14–21] or strains artificially selected in laboratory [6, 22–25]. Resistance to the Bin toxin can be due to mutations in the *cqm1/cpm1* gene encoding the toxin receptor, which prevent the expression of Cqm1/Cpm1 proteins that are bound on the midgut epithelium by its GPI anchor [26–31]. The *cqm1*_REC_ allele was identified in a Bin-resistant *Cx. quinquefasciatus* strain (REC) derived from Recife city (Brazil) that was subjected to artificial selection in our insectary, and it is the major gene that confers the resistance of this strain [23]. This allele has a 19-nucleotide (nt) deletion that encodes a truncated protein without the GPI anchor; therefore, the Cqm1 receptor is no longer available bound to the cell membrane [27, 31]. DNA-screening in field population detected the *cqm1*_REC_ allele in six nontreated areas, with frequency between 1 and 6 × 10^–3^, and in one *L. sphaericus*-treated area at a higher frequency (5 × 10^–2^) [26, 32]. The *cqm1*_REC_ is recessively inherited and only the homozygous individuals are resistant to the Bin toxin [6, 27]. The Bti crystal has a complex action on multiple receptors, and to date, there are no reports of resistance to the Bti crystal in field populations subjected to treatments [4, 33] or strains submitted to artificial selection [34–36]. The complex action of the set of toxins from the Bti crystal is a determining factor to prevent the onset of resistance. The selection in laboratory can lead to resistance but only when single Bti toxins were used, that is, not when using the whole crystal [36, 37]. The major limitation of Bti crystal is its vulnerability to biotic and abiotic factors, which makes its field persistence shorter, in particular under solar radiation [1, 38].

Thus, combined larvicides containing crystals with both protoxins from *L. sphaericus* and Bti, here named *L. sphaericus/*Bti, and also referred as long-lasting microbial larvicides [39–41], were developed to offer advantages as a broader spectrum of action for controlling different species occurring in the same breeding sites, improved field persistence, and low risk for selecting resistance. The utilization of *L. sphaericus/*Bti larvicides has been evaluated under different scenarios, in particular, for *Anopheles* in peri-urban [42–44] and rural areas that are endemic for malaria in Africa [39–41, 45, 46]. Their effectiveness for controlling anophelines in fish farming ponds and other breeding sites from malaria-endemic areas in Brazil has also been demonstrated [47]. In view of their environmentally safe profile, *L. sphaericus/*Bti larvicides can be used to control mosquitoes in sylvatic ecosystems and sensitive areas [48]. *L. sphaericus/*Bti larvicides have been used to fight *Culex* and *Aedes*, but most reports come from areas with relatively low proliferation of mosquitoes and pathogens [49–53]. The evaluation of *L. sphaericus/*Bti larvicides for controlling species such as *Cx. quinquefasciatus* and *Ae. aegypti* in areas characterized by their simultaneous and permanent proliferation in territories under high pathogen transmission is still scarce. Furthermore, few studies have compared the susceptibility of these target species with these compounds. Our hypothesis is that the association of mosquito-active protoxins in *L. sphaericus*/Bti-based larvicides can display high efficacy against *Cx. quinquefasciatus*, including the Bin-resistant larvae, and to *Ae. aegypti* that can be found in the same territory of urban areas. Therefore, this study aimed to determine the susceptibility of these species to a *L. sphaericus*/Bti larvicide under laboratory conditions and to determine its persistence for controlling these larvae in the same breeding site under simulated field trials. In addition, we evaluated whether the *L. sphaericus*/Bti larvicide could reduce the frequency of *Cx. quinquefasciatus* Bin-resistant larvae, using a laboratory colony established with a known frequency of resistant genotypes.

## Methods

### Mosquito colonies

Three colonies kept in the insectary of Instituto Aggeu Magalhães (IAM-Fiocruz) were used. The *Cx. quinquefasciatus* CqSLab, here named CqS, is a susceptible reference colony established with eggs collected in Recife city (Brazil) whose individuals are homozygous for the *cqm1* allele that encodes the receptor of the Bin toxin [31, 54]. The *Cx. quinquefasciatus* REC, here named CqR, displays high resistance ratio (RR > 1000-fold) to the Bin toxin from *L. sphaericus* strain 2362 and is composed of homozygous individuals for the *cqm1*_*REC*,_ the major recessive allele that confers Bin-resistance [27, 55]. The *Cx. quinquefasciatus* SREC colony was established for this study, using CqS and CqR individuals to evaluate the genotypes for the *cqm1* and *cqm1*_*REC*_ alleles, after larvae were subjected to larvicides treatments for four generations, as further described in the section Establishment, maintenance, and treatment of the SREC colony. The *Ae. aegypti* Rockefeller colony, here named Rocke, is an international reference for insecticide susceptibility and was used in this study. All colonies were maintained under controlled insectary conditions of temperature (26 ± 1 °C), relative humidity (70%), and photoperiod (14 h light:10 h dark). Larvae were reared in tap water from the public supply system and fed cat food (Friskies®). The adults fed sucrose solution 10% ad libitum, and females also fed, once per week, on defibrinated rabbit blood provided by the Institute of Science and Technology in Biomodels- Fiocruz (Rio de Janeiro, RJ, Brazil).

### Larvicides

Two microbial larvicides from Sumitomo Chemical/Valent Biosciences (www.valentbiosciences.com/publichealth/products) were used. The combined *L. sphaericus*/Bti based-larvicide was VectoMax FG™ (batch 313–533-N830) containing 2.7% of crystals/spores from *L. sphaericus* 2362 (strain ABTS 1743) and 4.95% of crystals/spores from Bti (strain AM65-52) as active ingredients, with a potency of 50 *L. sphaericus* international toxic units (ITU)/mg against *Cx. quinquefasciatus* larvae. The doses of VectoMax FG™ recommended by the manufacturer are the following: for *Culex* spp (open areas 5–20 kg/ha, cesspits 5–10 g/m2, polluted water 10–20 kg/ha); for *Anopheles* spp (open areas 5–10 kg/ha); and for *Aedes* spp (open areas 5–10 kg/ha, water reservoirs 2–4 g/100 L, polluted water 20 kg/ha). The larvicide VectoLex WG® (batch 285–416-PG30), containing only 51.2% of crystals/spores from *L. sphaericus* 2362 (strain ABTS 1743) with a potency of 650 *L. sphaericus* ITU/mg against *Cx. quinquefasciatus* larvae, was used for the selection of Bin-resistant *Cx. quinquefasciatus* larvae from the SREC colony, or for comparative purposes, when needed. Both larvicides are presented as slow-release granules and were stored at room temperature (RT) according to the manufacturer’s instructions. Additional technical information is available at sumitomochemical.com/ehd-public-health-products.

### Dose response bioassays

These assays were done to assess larvae susceptibility and to determine the lethal concentrations of *L. sphaericus*/Bti larvicide (Vectomax FG™) for 50% (LC_50_) and 90% (LC_90_) to groups of 20 third instar larvae in 100 ml of water, after 48 h exposure, which were performed on the basis of the standard protocol [55]. For these bioassays, stock aqueous suspensions using each larvicide were prepared on the basis of an adapted protocol at 5 g/L considering the content of the active ingredient (*L. sphaericus*/Bti 7.65%). Then, the stock suspension at 5 g/L was incubated at RT for 72 h under gentle agitation to release the active ingredients. After, the samples were subjected to agitation (Vortex) for 3 min at RT. Immediately after this step, the liquid phase was collected to prepare aliquots that were stored at −20 °C, until use. The solid phase was discarded. The dose–response bioassays were performed using experimental sets of 20 late third instar larvae in cups with 100 mL of distilled water, in three biological replicas, and food was not provided. In each bioassay, three technical replicas of larvae were exposed to each of six concentrations of the bacterial suspensions (0.0025–0.1 mg/L) diluted from the stock suspension (5 g/L). An untreated control group, three technical replicas of larvae, was run at each bioassay. The mortality was recorded after 48 h and the maximum mortality allowed in the untreated control was 10%. A total of three or four biological replicas of each bioassay were done to determine the LC_50_ and LC_90_ values and their respective 95% confidence interval (CI) on the basis of probit analysis using SPSS v.16.0 for Windows. The LCs whose 95% confidence intervals (CI) overlap are not considered different.

### Diagnostic concentrations bioassays

Bioassays were also done to empirically determine the concentrations of *L. sphaericus*/Bti (Vectomax FG™) and *L. sphaericus* (VectoLex WG®) that could be lethal to around 80–90% of large pools of third instar larvae (*n* = 100–300) set in rearing trays filled with tap water (1 L) and a small amount of food (0.05–0.1 g/tray). Pools of larvae from the CqS, CqR, and Rocke strains were tested. The stock suspension at 5 g/L were prepared as described in the section Dose response bioassays, considering the content of the active ingredient of each larvicide tested (*L. sphaericus*/Bti 7.65%, *L. sphaericus* 52.1%). Single concentrations between 0.005 mg/L and 0.2 mg/L were tested against pools of larvae of each strain. At least three technical replicas per concentration tested and per strain were used. The mortality was recorded after 48 h exposure. These assays indicate the concentrations of each larvicide that were used to treat the third instar of the SREC larvae at every generation.

### Simulated field trial

**Figure 1:**
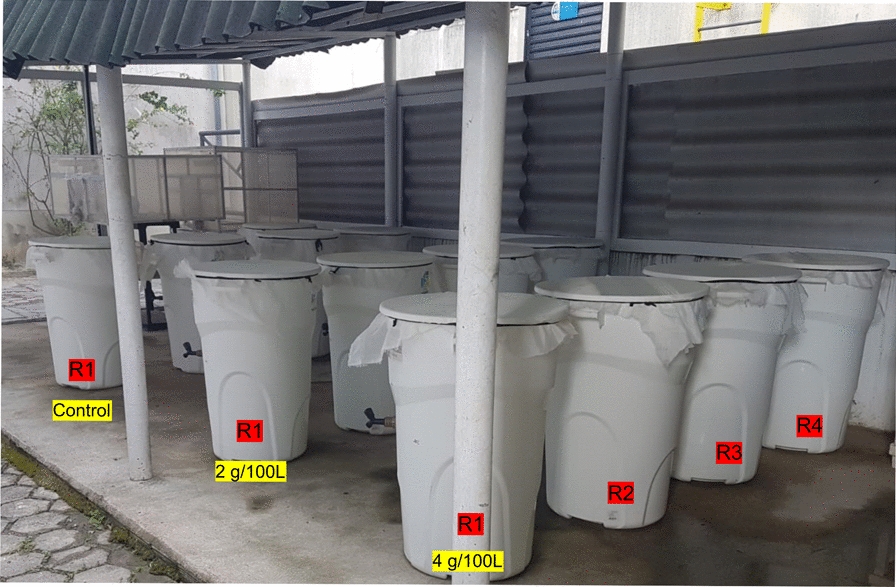
Experimental area of the trial for evaluating the residual activity of a larvicide to mosquito. *Lysinibacillus sphaericus*/*Bacillus thuringiensis* svar. *israelensis* larvicide (Vectomax™ FG) was tested against third instar larvae of *Culex quinquefasciatus* and *Aedes aegypti* (*n* = 150) in containers with 100 L of water treated with 2 g/container or 4 g/container using four replicas (R). An untreated control group was run during the trial

A simulated field trial to evaluate the residual activity of the combined *L. sphaericus*/Bti larvicide against *Cx. quinquefasciatus* and *Ae. aegypti* larvae colonizing the same breeding site was run in an experimental area at IAM-FIOCRUZ from October 2022 to January 2023. This facility was covered on top and open on the sides, and containers, which simulated larvae breeding sites, were not exposed to rain or direct sunlight (Fig. 1). The methodology, adapted from previous studies [56, 57], is briefly described here. The breeding sites were 150 L-white plastic containers filled with 100 L of tap water, 0.1 g of food, and colonized with a pool of 150 third instar larvae composed of CqS (*n* = 70), CqR (*n* = 30), and Rocke (*n* = 50). All containers were covered with a fine mesh. The *L. sphaericus*/Bti larvicide was tested at 2 g or 4 g per container with 100 L of water, using four technical replicas. These doses were based on the manufacturer’s recommendation for *Aedes* in water reservoirs, as described in the section Larvicides. An untreated control group using four technical replicas was kept during the trial. After the first colonization with larvae, the containers, except the control ones, were treated. The larvae mortality in the experimental containers was evaluated 48 h after the colonization done each week, during 12 weeks. The dead larvae were not removed from the containers. The persistence in the treated groups was considered suitable when the average mortality in the treated containers was ≥ 80%. When the mortality in the untreated containers exceeded 20%, the container was excluded and replaced. The temperature and relative humidity were recorded weekly during the trial.

### Establishment, maintenance, and treatment of the SREC colony

The SREC colony was established with CqS and CqR adults with the purpose of evaluating the genotypes for the *cqm1* and *cqm1*_*REC*_ alleles of individuals, after treatments with the larvicide, along four generations. Briefly, the major procedures carried out in each generation, represented in the Additional file 1 (Fig. S1), were: determination of the genotypes of a larvae sample before the treatment, treatment of larvae, mortality recording, and determination of the genotype in a sample of the surviving adults. The parental generation was composed of 200 adults (1:1 sex ratio) using a frequency of 0.70 of CqS (*n* = 140, 1:1 sex ratio) and 0.30 of CqR (*n* = 60, 1:1 sex ratio). Pupae from each sex and each strain were kept separately in individual cages. After emergence, adults from those four cages were pooled simultaneously in a single cage (30 cm × 30 cm × 30 cm) as an effort to promote a random crossing. Adults were kept as previously described. After the first gonadotrophic cycle, each filial generation was established using egg samples, taken from around 76 rafts, and larvae were reared under controlled conditions [54]. Briefly, groups of 500–700 larvae were reared in trays (34 cm × 24 cm × 7 cm, 4 L capacity) filled with 2 L of tap water and 1.6 g of cat food provided during the larval phase. When the larvae achieved the third instar, the genotype of a larvae sample was determined (*n* ~ 50–100) by polymerase chain reaction (PCR) assays, as described in the next section. After this step, between 1200 and 3600 third instar larvae per generation were treated with a concentration of larvicide that was established in the section Diagnostic concentrations bioassays. After the treatment, the surviving larvae were washed twice with tap water, transferred to trays, and reared under standard conditions until they reached the adult stage. The F_1_ larvae were treated with *L. sphaericus* to promote the selection of Bin-resistant individuals, while F_2_, F_3_, and F_4_ larvae were treated with *L. sphaericus*/Bti larvicide. Total mortality, achieved until the emergence of adults, was recorded and the genotype of those individuals was also assessed (*n* ~ 50). This assay was performed twice.

### PCR assays

The genotype for the *cqm1* and *cqm1*_REC_ alleles of *Cx. quinquefasciatus* individuals from the SREC colony was determined using a PCR assay [32]. The 19-nt deletion found in the allele *cqm1*_REC_ was previously reported [31] and the full-length cDNA sequence is deposited in Gene Bank (accession number DQ333335). The individuals were classified for this genotype as: susceptible (*cqm1*/*cqm1* or SS, *cqm1*/*cqm1*_REC_ or SR) and resistant (*cqm1*_REC_/*cqm1*_REC_ or RR) according to the amplicon sizes, as described below. The PCR assay was designed to amplify a fragment from the *cqm1* gene using two primers that flank the 19-bp deletion found in the *cqm1*_REC_ allele, producing amplicons of 208 bp or 189 bp from the *cqm1* and *cqm1*_REC_ alleles, respectively [31]. At each generation, larvae and adult samples were collected and individually stored at −80 °C for genotype determination. The DNA extraction from these samples was done using DNAzol™ (Invitrogen, Carlsbad, CA, USA) according to the manufacturer’s recommendations, and procedures to prevent DNA contamination were also adopted. The DNA samples were quantified using a NanoDrop 2000c® spectrophotometer (Thermo Fisher Scientific), and concentration was normalized to 12.5 µg/µl. The PCR reactions were carried out as described [32] using forward primer (5'-CGAGAATTCATGCAGGACTTCAAAGAG-3’) and reverse primer (5'-GCACTGCAGGGAAGTGGTGGAAGGTAC-3). The amplified products were separated by electrophoresis in a 2.5% agarose gel, stained with ethidium bromide, and visualized in an ultraviolet transilluminator. The following controls were run during each reaction: a positive control with DNA from a known homozygous-susceptible individual to amplify the 208 bp amplicon; a positive control with DNA from a known resistant individual to amplify the 189 bp-amplicon; and a negative control sample without DNA. To confirm the identity of the amplicons, 17 samples of PCR products were purified using the Qiaquick® PCR & Gel Cleanup Kit (QIAGEN, Hilden, Germany) and then subjected to sequencing with the ABI PRISM® 3100 Genetic Analyzer (Applied Biosystems, Foster City, CA, USA) at the Núcleo de Plataformas Integradas (NPT) from IAM- Fiocruz.

## Results

### Toxicity to larvae

The combined *L. sphaericus*/Bti larvicide showed high activity against all three strains tested, which was decreasing for CqS, CqR, and Rocke larvae (Table 1). The LCs values presented in Table 1 represent the average of the LCs obtained in three or four independent bioassays and the 95% confidence intervals represent the range of the intervals found in those assays (Table S1). The average LC_50_ and LC_90_ of *L. sphaericus*/Bti toward CqS larvae were 0.006 mg/L and 0.030 mg/L, while the respective LCs for CqR larvae were 0.009 mg/L and 0.069 mg/L. At the LC_90_, only a 2.3-fold increase toward the CqR larvae compared with the CqS was found. This larvicide also showed high toxicity to Rocke larvae, which is naturally refractory to *L. sphaericus* Bin toxin, with an LC_50_ of 0.042 mg/L and LC_90_ of 0.086 mg/L. The comparison of the toxicity ratio, taking the most susceptible larvae CqS as the reference, showed that the larvicide acts on the CqR and Rocke larvae with a LC_90_, which was only threefold greater. The TR at LC_90_ between CqR and CqS is not considered significant, as the 95% confidence intervals (CI) overlap, while the TR at LC_90_ between Rocke and CqS was considered significant. A total of 11 bioassays comprising a total of 3900 larvae were used to establish the LCs and the analytical data generated from each bioassay is presented in the Additional file 2: Table S1.

**Table 1:** Toxicity of a combined *Lysinibacillus sphaericus*/*Bacillus thuringiensis* svar. *israelensis* (Vectomax™ FG) larvicide to mosquito larvae

Strain^1^	No. larvae	LC_50_ (95% CI) ^2^	TR^3^	LC_90_ (95% CI)	TR
CqS	1140	0.006 (0.003–0.011)	1.0	0.030 (0.012–0.063)	1.0
CqR	1260	0.009 (0.005–0.017)	1.2	0.069 (0.023–0.269)	2.3
Rocke	1500	0.042 (0.032–0.053)	7.0	0.086 (0.064–0.131)	2.9

^1^ Third instar larvae of *Culex quinquefasciatus* (CqS), *Cx. quinquefasciatus* resistant to the Binary toxin (CqR), and *Aedes aegypti* Rockefeller (Rocke). ^2^ Lethal concentrations (mg/L) for 50–90% of larvae after 48 h of exposure and 95% confidence intervals (CI). ^3^ Toxicity ratio between the LC to CqR or Rocke larvae and the reference CqS

Toxicity of *L. sphaericus*/Bti and *L. sphaericus* larvicides was tested against larger pools of larvae from all strains set in rearing trays and results are presented in the Additional file 3: Table S2. Briefly, for CqS larvae, more than 80% mortality was achieved using from 0.02 mg/L of *L. sphaericus*/Bti and from 0.005 mg/L of *L. sphaericus* larvicide. For CqR larvae, 0.1 mg/L of the *L. sphaericus*/Bti provoked more than 90% mortality, while 0.1 mg/L of *L. sphaericus* did not provoke mortality, as was expected since these are Bin-resistant larvae. For Rocke, a mortality greater than 90% was observed using 0.2 mg/L of *L. sphaericus*/Bti, while larvae treated with 0.2 mg/L of *L. sphaericus* showed no mortality, as detected for CqR. This evaluation showed decreasing susceptibility for CqS, CqR, and Rocke larvae to *L. sphaericus*/Bti, similar to the pattern found in the dose-mortality bioassays. The results also confirmed the susceptibility of CqS to *L. sphaericus* larvicide, contrasting with the resistant profile of CqR and Rocke larvae to this compound.

### Persistence in a simulated field trial

**Figure 2:**
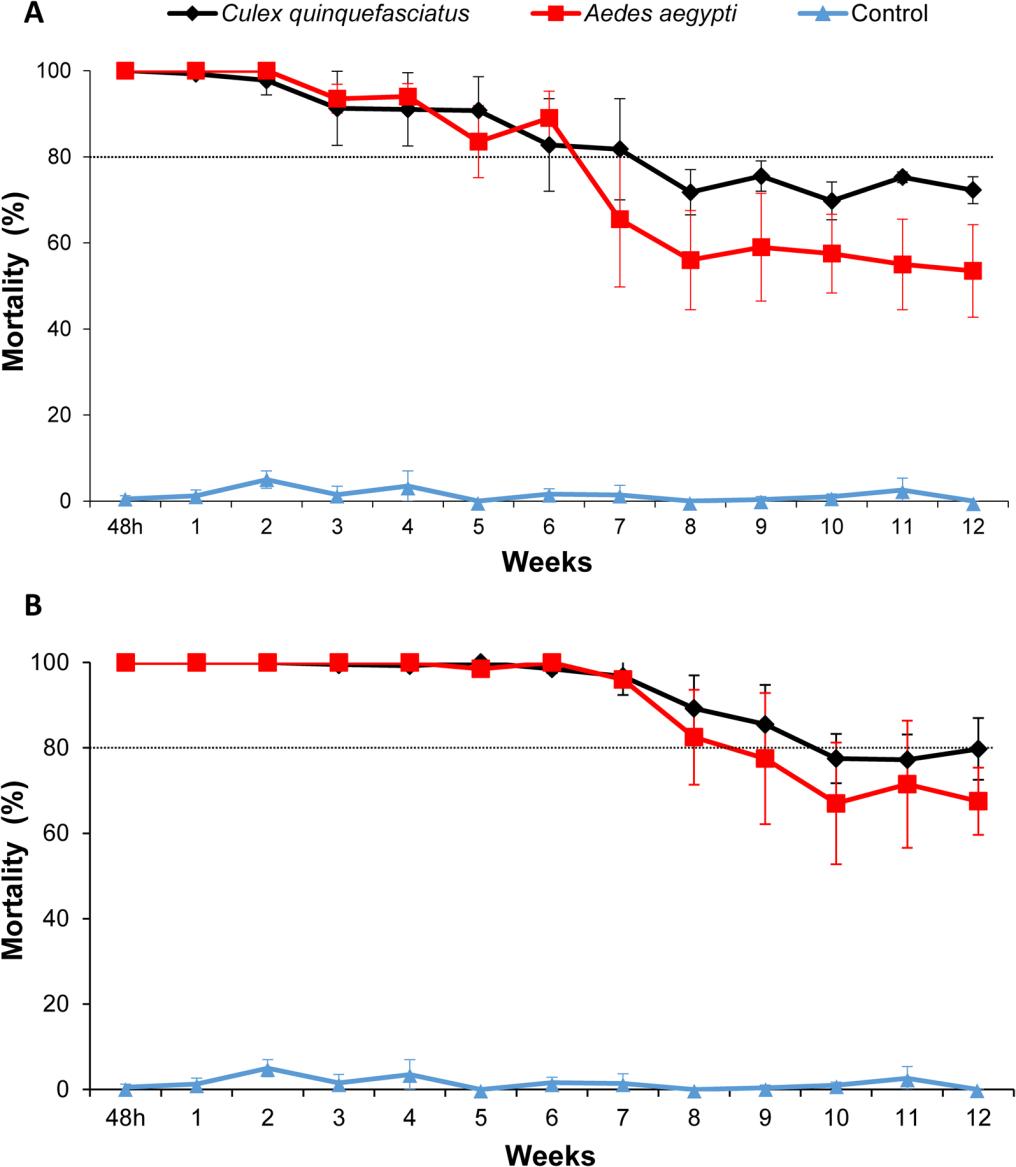
Residual activity of *Lysinibacillus sphaericus*/*Bacillus thuringiensis* svar. *israelensis* larvicide (Vectomax™ FG) for mosquito larvae. This trial was performed under simulated field conditions using samples of third instar larvae of *Culex quinquefasciatus* strain CqS (*n* = 70), *Cx. quinquefasciatus* resistant to the Binary toxin strain CqR (*n* = 30), and *Aedes aegypti* strain/Rockefeller (*n* = 50). At every week, larvae samples (*n* = 150) were introduced into each container with 100 L of water, and the average mortality in the four replicates of the treated and untreated groups (control) was recorded 48 h after each colonization. **(A)** Treatment with 2 g/100 L. **(B)** Treatment with 4 g/100 L

The residual activity of *L. sphaericus*/Bti-larvicide (Vectomax™ FG) for controlling CqS, CqR, and Rocke larvae cohabiting the same breeding site was evaluated under simulated field conditions. This trial took place for 12 weeks during the warm season of Recife city, with temperatures of 29.1 ± 1.5 °C (27.9 ± 1.0 °C in the water of containers) and relative humidity ranging between 52% and 88%. Treatment with *L. sphaericus*/Bti-larvicide at 2 g/container or 4 g/container showed initial efficacy, as 100% mortality for all larvae groups was recorded 48 h after the single treatment (Fig. 2). The persistence, considering an average mortality ≥ 80% for all larvae, was 6 weeks and 8 weeks using 2 g or 4 g/container, respectively (Fig. 2). The persistence according to the species was greater for *Cx. quinquefasciatus*, 7 weeks and 9 weeks at 2 g/container and 4 g/container, respectively, than for *Ae. aegypti* larvae, 6 weeks and 8 weeks at 2 g/container and 4 g/container, respectively. After 12 weeks, when the trial was finished, 72% and 54% average mortality for *Cx. quinquefasciatus* and *Ae. aegypti* larvae, respectively, was still recorded in the set containers treated with 2 g. In the containers treated with 4 g, 80% and 68% of mortality for the respective species mentioned above was detected. Overall, the finest performance for both species was achieved using 4 g/container, which provided an average mortality above 80% for 8 weeks and remained around 65% after 12 weeks. The analysis of the replicate dataset showed an earlier mortality decline in the R1 containers (Additional file 4: Table S3, Additional file 5: Fig. S2), which were exposed to a greater indirect solar incidence compared with the others due to their position in the experimental area (Fig. 1). In the containers treated with 2 g/container, the mortality of both species in the R1 declined below 80% in the third week. For those treated with 4 g, a similar decline was observed in the R1 container after 8 weeks. During the trial, some replicates of the untreated control group showed mortality greater than 20%; when this occurred, the container was replaced.

### Genotypes of the SREC individuals treated with larvicides

**Figure 3:**
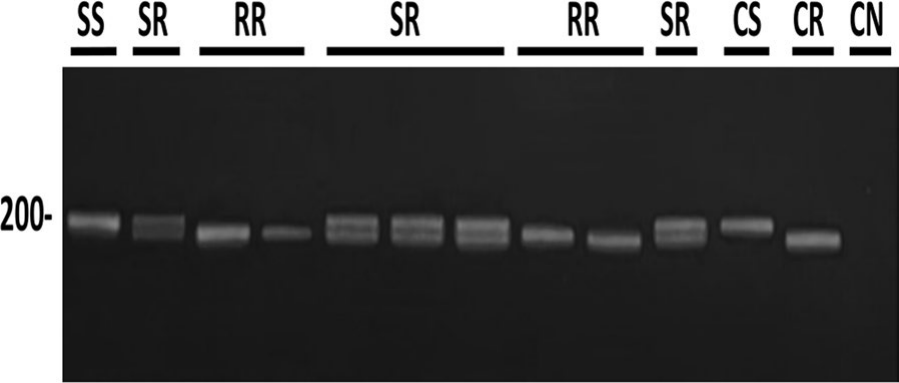
Fragments of *Culex quinquefasciatus cqm1* (208 bp) and *cqm1*_*REC*_ (189 bp) alleles amplified by PCR. The pProfile of amplicons produced are: homozygous-susceptible *cqm1/cqm1* (SS), homozygous-resistant *cqm1*_*REC*_*/cqm1*_*REC*_ (RR), and heterozygous-susceptible *cqm1/cqm1*_*REC*_ (SR) individuals. CS. Positive control for the *cqm1* amplicon (208 bp). CR. Positive control for the *cqm1*_*REC*_ amplicon (189 bp). CN. Negative control sample without DNA. The DNA used in the test samples was extracted from larvae of the SREC strain (F_4_). Molecular size marker of 200 bp on the left

**Figure 4:**
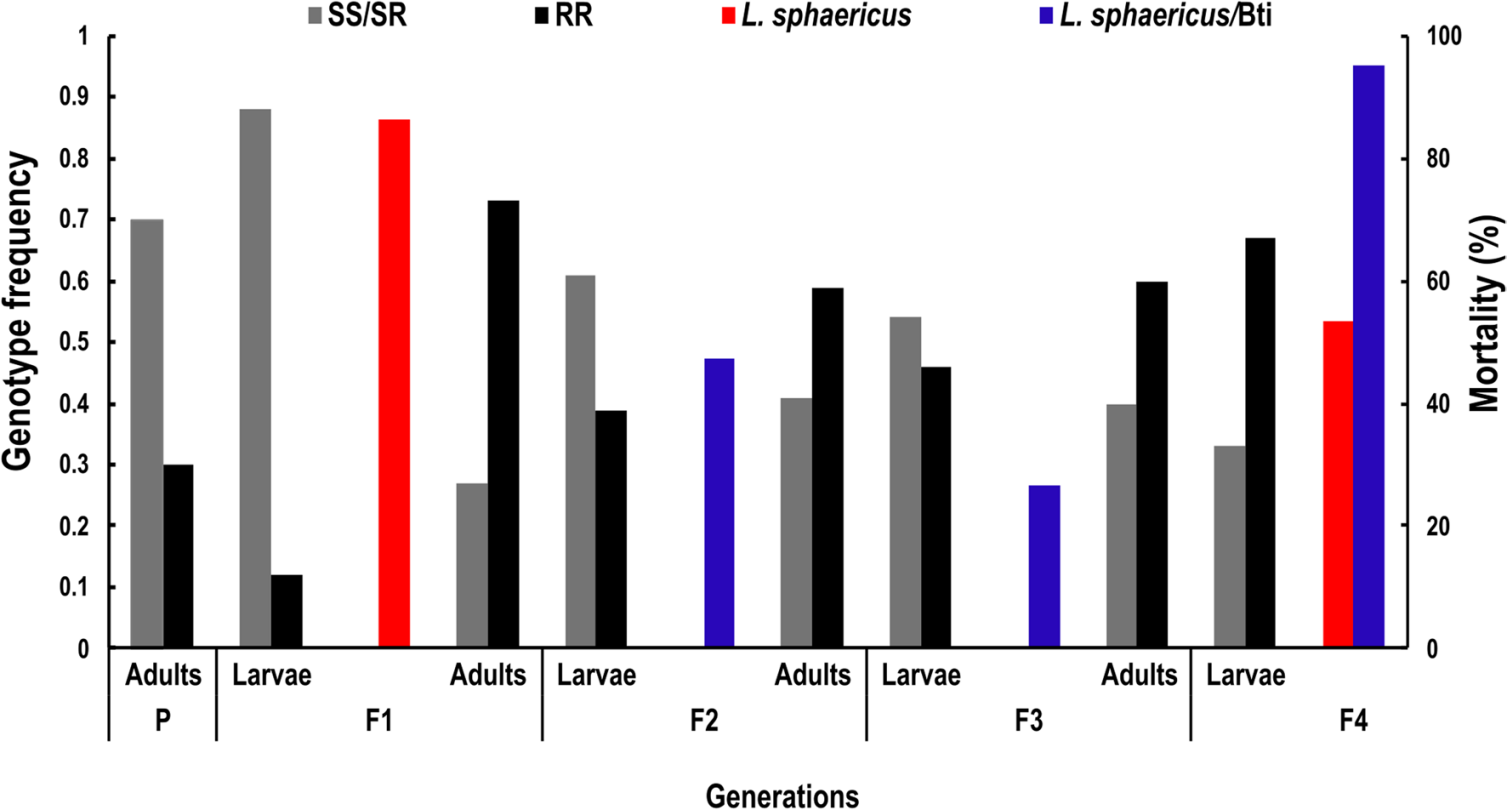
Genotypes for *cqm1* and *cqm1*_*REC*_ alleles in individuals from the *Culex quinquefasciatus* SREC strain. The parental generation (P) was established with homozygous-susceptible and homozygous-resistant adults. The genotypes of larvae were determined in samples (*n* ~ 100) at each generation (F) before treatment. Larvae from each generation were treated with *Lysinibacillus sphaericus-*VectoLex WG® (F_1_) or *L. sphaericus*/*Bacillus thuringiensis* svar. *israelensis-*VectoMax FG™ (F_2_-F_4_). Mortality was recorded and the genotypes of a sample of surviving adults (*n* ~ 50) was assessed. Full dataset is available in Table 2 and Additional file 7: Table S4

The genotypes of the SREC individuals for the *cqm1* and *cqm1*_REC_ alleles, subjected to treatments with *L. sphaericus* or *L. sphaericus*/Bti larvicides, were determined by PCR (Fig. 3) across four generations. Samples of amplicons with the observed sizes of 208 bp (*n* = 10) and 189 bp (*n* = 7) were sequenced, and their identity, as fragments of the *cqm1* and *cqm1*_REC_ alleles, respectively, was confirmed (Additional file 6: Fig. S3). The results are presented in Table 2 and Fig. 4, and the mortality dataset is available in Additional file 7: Table S4. First, the genotypes of samples of CqS (*n* = 10) and CqR (*n* = 10) larvae that were used for the parental generation were assessed, and they displayed the expected SS and RR genotypes, respectively. This parental generation (SS 0.7, RR 0.3) produced F_1_ larvae that displayed a reduction of the frequency of the resistant ones (SS 0.6, SR 0.3, RR 0.1), and this dilution effect was observed at each progeny. The treatment of F_1_ larvae with *L. sphaericus* (86% mortality) resulted in the increase of the RR frequency in the surviving adults (0.7). For F_2_ larvae, the RR genotype decreased as expected (RR 0.4). These larvae were treated using *L. sphaericus*/Bti with a lethal concentration of around 70% for susceptible larvae (0.01 mg/L); only 47% mortality was achieved and the RR genotype increased for the surviving adults (0.6). This *L. sphaericus*/Bti concentration was used to treat F_3_ larvae and similar results were obtained: low mortality and the rise of RR frequency among adults (0.6). Those results showed that sublethal doses of *L. sphaericus*/Bti to treat larvae samples that exhibit a high frequency of RR genotype have a selective effect. In view of these results, the F_4_ larvae with a high RR frequency (0.7) were then treated with a concentration of *L. sphaericus*/Bti lethal for 90% of resistant larvae (0.1 mg/L), and 95% mortality was achieved. In parallel, another sample of F_4_ larvae treated with *L*. *sphaericus* at the same concentration (0.1 mg/L) showed only 45% mortality. These results showed that *L. sphaericus*/Bti eliminate resistant individuals when suitable concentrations were used. Positive control samples using SS larvae treated with *L*. *sphaericus*/Bti or *L. sphaericus* larvicides at 0.1 mg/L displayed 99% mortality for both larvicides (Additional file 7: Table S4). This assay was performed again using another parental strain named SREC2 established with the same initial frequency of the resistant allele, and produced similar results that were recorded during three generations (Additional file 8: Table S5, Additional file 9: Table S6, Additional file 10: Fig. S4).

**Table 2:** Genotypes for the *cqm1* and *cqm1*_*REC*_ alleles in individuals from the *Culex quinquefasciatus* SREC strain

		Frequency in larvae								Frequency in adults						
			Genotype				Allele				Genotype				Allele	
Generation		No	SS	SR	RR		S	R		No	SS	SR	RR		S	R
P		NA	NA	NA	NA		NA	NA		30	0.70	0.0	0.30		0.70	0.30
F_1_		96	0.60	0.28	0.12		0.74	0.26		48	0.19	0.08	0.73		0.23	0.77
F_2_		88	0.27	0.34	0.39		0.44	0.56		42	0.24	0.17	0.59		0.32	0.68
F_3_		95	0.20	0.34	0.46		0.37	0.63		48	0.15	0.25	0.60		0.27	0.73
F_4_		95	0.08	0.25	0.67		0.20	0.80		NA	NA	NA	NA		NA	NA

Genotypes: susceptible homozygous (SS), susceptible heterozygous (SR), and resistant homozygous (RR). Parental generation (P) was set with SS and RR adults. At each generation (F) larvae were treated and mortality was recorded (Additional file 7: Table S4). Genotypes of larvae were determined before treatment and genotypes of surviving adults were also assessed. NA, not applicable

## Discussion

The toxicity of combined *L. sphaericus*/Bti larvicides to different target mosquito species has been scarcely assessed when compared with data available for those individual larvicides. Our study demonstrated that the *L. sphaericus*/Bti larvicide (Vectomax™ FG) displayed high toxicity to *Cx. quinquefasciatus*, including Bin-resistant *Cx. quinquefasciatus* and *Ae. aegypti*. This larvicide showed action for these individuals lacking functional receptors for the Bin toxin in the midgut [5, 7, 27] whose LC_90_ value was higher, but it was only threefold compared with that for *Cx. quinquefasciatus* CqS, considered as the reference strain. Indeed, previous studies showed that Bin-resistant *Cx. quinquefasciatus* and *Ae. aegypti* larvae were susceptible to Bti [23, 58–60] and to mixtures of Bin and Cyt1Aa toxin [59, 61–64]. This is likely due to the Cyt1Aa toxin from Bti that enables the Bin toxin to enter the midgut epithelial cells lacking Bin-receptors, whose mechanism is related to the ability of Cyt1Aa to form pores in the cell membrane allowing the entry of Bin [65]. It is worth noting that the LCs found in the present study, and in a previous one, suggest that the Bin action in the midgut cells lacking Bin-receptors might be less efficient compared with that in which the Bin toxin interacts with those cells having Bin-receptors, since the LC values are lower [65]. Nevertheless, the most important fact is that Cyt1Aa synergizes not only the Bti Cry toxins, but also the Bin toxin, improving the in vivo toxicity.

Comparing our toxicity data of *L. sphaericus*/Bti to *Cx. quinquefasciatus/Cx. pipiens* and to *Ae. aegypti* with few reports that are available, our LC_90_ values were much lower [19, 20, 66, 67]. This may be explained by the origin and rearing conditions of the tested larvae, but also to the methodological procedures to process the stock suspensions for the bioassays. This is particularly relevant when testing commercial products. In our study, for instance, it is likely that the step of incubation for releasing the active ingredient from the Vectomax™ FG granules could be behind the greater toxicity found. The LCs values of Vectomax™ FG to *Aedes albopictus* [68], previously established, were close to those found for *Ae. aegypti* in our study, and both were determined using the same methodology. Therefore, the evaluation of the toxicity (LCs) of commercial products, instead of technical powders, must be carefully analyzed to avoid mistaken conclusions. From a qualitative point of view, our dataset is in agreement with in vivo toxicity studies from the literature, as they revealed the same pattern of decreasing susceptibility of *Cx. quinquefasciatus* and *Aedes* spp. This is an important parameter for establishing suitable doses for treating different species that can be found in same breeding sites.

The coexistence and high proliferation of *Cx. quinquefasciatus* and *Ae. aegypti* larvae in the same areas [69] and even in the same breeding sites [70, 71], might reflect the scenario of several urban areas in endemic countries. In our study, the performance of *L. sphaericus/*Bti larvicide in containers colonized with both *Cx. quinquefasciatus* and *Ae. aegypti* larvae showed promising results with a persistence of 8 weeks, providing at least 80% mortality to both species after a single treatment. The greater persistence observed toward *Cx. quinquefasciatus* compared with *Ae. aegypti* corroborates the susceptibility profile found for these species in the bioassays. In addition, it is possible that the lower persistence to *Ae. aegypti* recorded under simulated field conditions could also be attributed to the faster degradation of Bti crystals, mainly due to temperature and solar radiation compared with the *L. sphaericus* crystals [56, 72–74]. Under such conditions, the lower availability of Cyt1Aa toxin, which is necessary to synergize the action of Bin and Cry toward *Ae. aegypti* [59, 65], could, for instance, reduce the toxicity to *Ae. aegypti* larvae. The 8-week persistence period recorded for the *L. sphaericus/*Bti larvicide (Vectomax™ FG), under the conditions tested, is consistent with other trials in urban and peri-urban for controlling *Culex* and *Aedes*, whose persistence ranged from 2 weeks to 5 weeks, according to the breeding sites (for example, catch basis, vegetated ditches), species, and other factors [53, 75–77]. The performance found for controlling anophelines, such as *Anopheles darlingi*, *Anopheles funestus*, and *Anopheles arabiensis*, in shaded areas and non-shaded areas was shorter, ranging from 1 week to 4 weeks [47, 78, 79]. The *L. sphaericus/*Bti product label informs a persistence up to 4 weeks, according to local conditions, and the greater persistence recorded in our trial could be related to the protection from the direct insolation and rain, since these are the main factors that negatively impact the persistence of microbial larvicides [1]. Our dataset revealed an earlier increase of the mortality in containers that were more exposed to indirect sunlight than the others, which corroborates this hypothesis. Another factor to be considered is that, in our trial, the dead larvae were not removed from the containers, and this condition might have allowed for the recycling of *L. sphaericus* and Bti spores [1, 56], providing an extended action. The removal of dead larvae used in some studies [79] might avoid the beneficial effects of recycling.

The detection of Bin-resistance alleles in *Cx. quinquefasciatus* populations from several countries remains a threat for the onset of resistance [4]. Our study, using the SREC colony as a model to assess the frequency of resistant genotype of larvae submitted to larvicide treatments, demonstrated that a single *L. sphaericus* treatment of larvae with a high frequency of that genotype could dramatically raise the frequency. As previously described, the frequency of the *cqm1*_*REC*_, which was the major resistance allele found in nontreated populations from Recife city, was low (10^–3^) [26, 32, 80]. Therefore, the selection of a resistance allele with a low initial frequency, associated with the recessive inheritance of the *cqm1*_REC_ [27], can be a gradual process. Further, in treated areas with records of operational failures, the frequency of such alleles in treated populations can be high [28, 30, 32] and their selection can be fast, as demonstrated in this study. In this scope, our results reinforced that the frequency and inheritance of the resistant alleles are key parameters for the resistance selection and can be used to indicate the adoption of proactive measures to avoid the rise of the resistance allele frequency [81]. Our data showed that once a high frequency of the Bin-resistant genotype was achieved, lethal doses of *L. sphaericus/*Bti larvicide to those individuals have to be used for their elimination, but sublethal doses can increase their frequency. *L. sphaericus/*Bti-based larvicides can also be employed to counteract the resistance to other insecticidal compounds. This was shown for an *Anopheles coluzzii* resistant to pyrethroids whose larvae were treated with *L. sphaericus*/Bti to eliminate resistant individuals and restore the susceptibility [82]. These results highlight the strategic importance of the complex composition of toxins in such larvicides for resistance management in different scenarios.

## Conclusions

Our dataset showed that the combined *L*. *sphaericus*/Bti larvicide displays high toxicity to *Cx. quinquefasciatus,* Bin-resistant *Cx. quinquefasciatus* and *Ae. aegypti*, and under a simulated trial it displayed a fine persistence compatible with bimonthly schemes of product application. The choice of suitable doses of the larvicide to control different target species considered their susceptibility profiles, which is crucial for its performance, as demonstrated for the control of *Cx. quinquefasciatus* and *Ae. aegypti*. These findings demonstrated that *L*. *sphaericus*/Bti larvicides can be an effective tool for controlling those species in urban areas with a low risk for selecting resistance.

## Supplementary Information

**Additional file 1: Figure S1**. Representation of the procedures for the maintenance of the *Culex quinquefasciatus* SREC strain.

**Additional file 2: Table S1**. Dataset of the dose–response bioassays of the *Lysinibacillus sphaericus*/*Bacillus thuringiensis* svar. *israelensis* against mosquito larvae.

**Additional file 3: Table S2**. Dataset of the diagnostic bioassays of the *Lysinibacillus sphaericus* and/or *Bacillus thuringiensis* svar. *israelensis* against mosquito larvae.

**Additional file 4: Table S3**. Dataset of the residual activity of *Lysinibacillus sphaericus*/*Bacillus thuringiensis* svar. *israelensis* larvicide toward mosquito larvae.

**Additional file 5: Figure S2**. Dataset of the residual activity of *Lysinibacillus sphaericus*/Bti to control mosquito larvae.

**Additional file 6: Figure S3**. Nucleotide sequences of amplicons of the *Culex quinquefasciatus cqm1* gene from SREC individuals.

**Additional file 7: Table S4.** Mortality of *Culex quinquefasciatus* larvae from the SREC strain from four generations (F).

**Additional file 8: Table S5.** Frequency of the genotypes for the *cqm1* and *cqm1*_*REC*_ alleles in *Culex quinquefasciatus* SREC2 strain.

**Additional file 9: Table S6.** Mortality of *Culex quinquefasciatus* larvae from SREC2 strain from four generations (F).

**Additional file 10: Figure S4.** Genotypes for the *cqm1* and *cqm1*_*REC*_ alleles in individuals from the *Culex quinquefasciatus* SREC2 strain.

## Data Availability

All data are provided in the manuscript or in the supplementary files.Raw data from all assays are available in the supplementary tables.

## Abbreviations

Bin: Binary toxin
Bti: *Bacillus thuringiensis* svar. *israelensis*
Cqm1: *Culex quinquefasciatus* maltase 1
*cqm1*: Gene encoding the *Culex* *quinquefasciatus* maltase 1
*cqm1*_*REC*_: Resistance allele of the *cqm1* gene
CqR: *Culex quinquefasciatus* Bin-resistant
Cqs: *Culex quinquefasciatus* susceptible
Fiocruz: Fundação Oswaldo Cruz
GPI: Glycosylphosphatidylinositol
IAM: Instituto Aggeu Magalhães
ITU: International toxic units
LC: Lethal concentration
Lsp: *Lysinibacillus sphaericus*
P: Parental generation
PCR: Polymerase chain reaction
REC: *Culex quinquefasciatus* strain composed of CqR individuals
Rocke: *Aedes aegypti* Rockefeller strain
RR: Homozygous individuals for the *cqm1*_*REC*_ allele
RR: Resistance ratio
RT: Room temperature
S: *Culex* quinquefasciatus strain composed of CqS individuals
SD: Standard deviation
SR: Heterozygous individuals for the *cqm1* and *cqm1*_*REC*_ alleles
SREC: *Culex quinquefasciatus* strain composed of CqS and CqR individuals
SS: Homozygous individuals for the *cqm1* allele
TR: Toxicity ratio
WHO: World Health Organization
