# Supplementary material for: Evaluation of a long-lasting microbial larvicide against Culex quinquefasciatus and Aedes aegypti under laboratory and a semi-field trial
Source: Parasit Vectors. 2024 Sep 14;17:391. doi: 10.1186/s13071-024-06465-5 (PMC11401406; doi:10.1186/s13071-024-06465-5)
Supplement: Supplementary file 1 — Additional file 1: Figure S1. Representation of the procedures for the maintenance of the Culex quinquefasciatus SREC strain. [file 13071_2024_6465_MOESM1_ESM.docx]

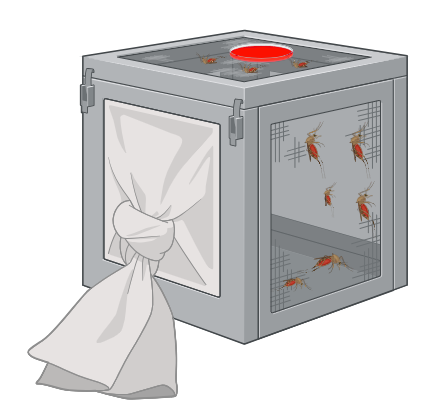

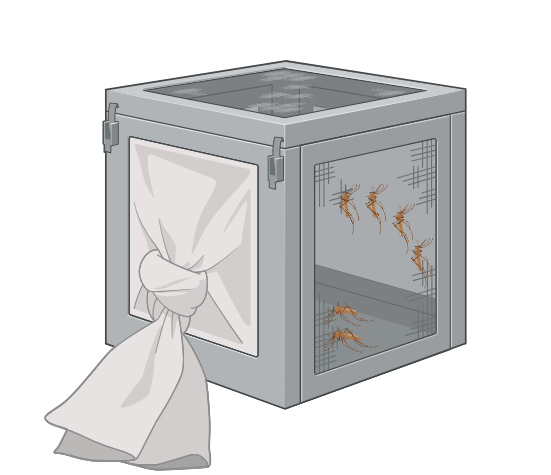

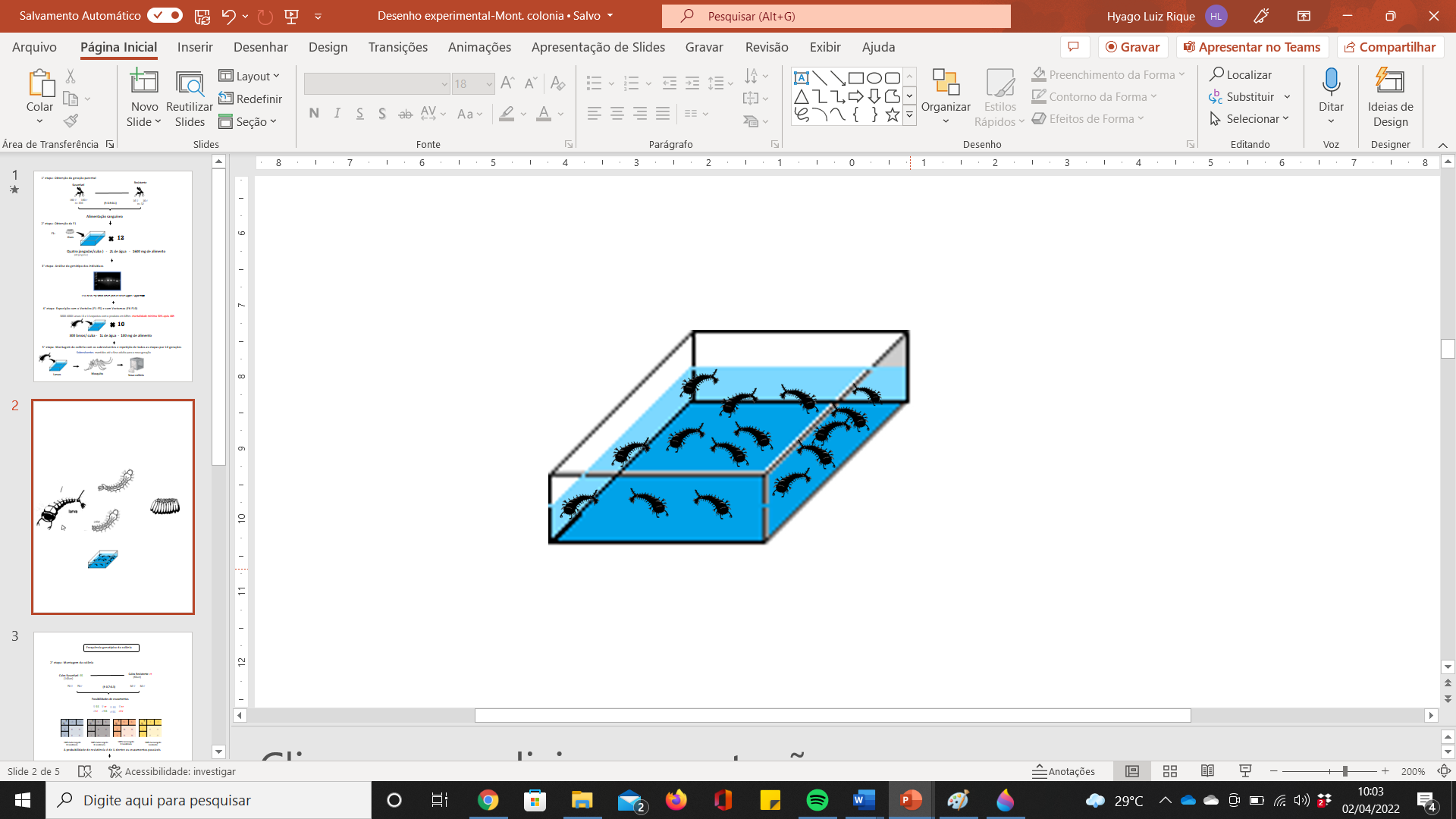


Parental


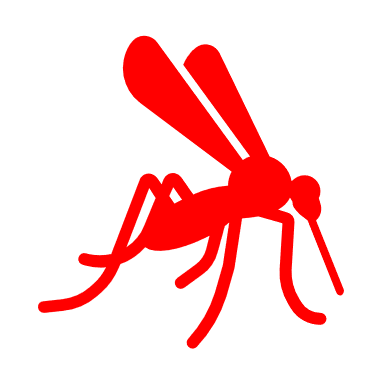

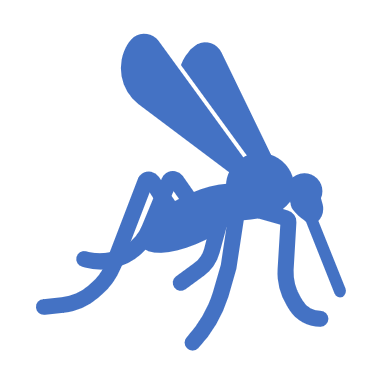


Susceptible

Resistant


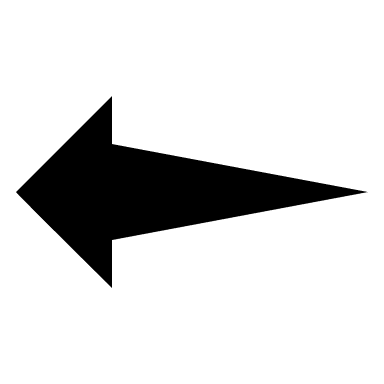

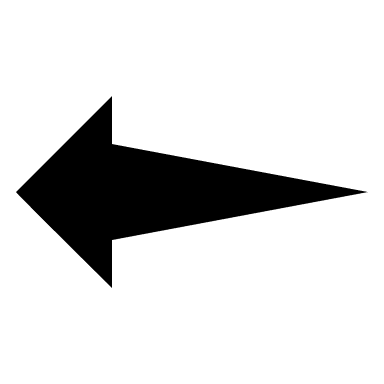


Larvae exposure-48h


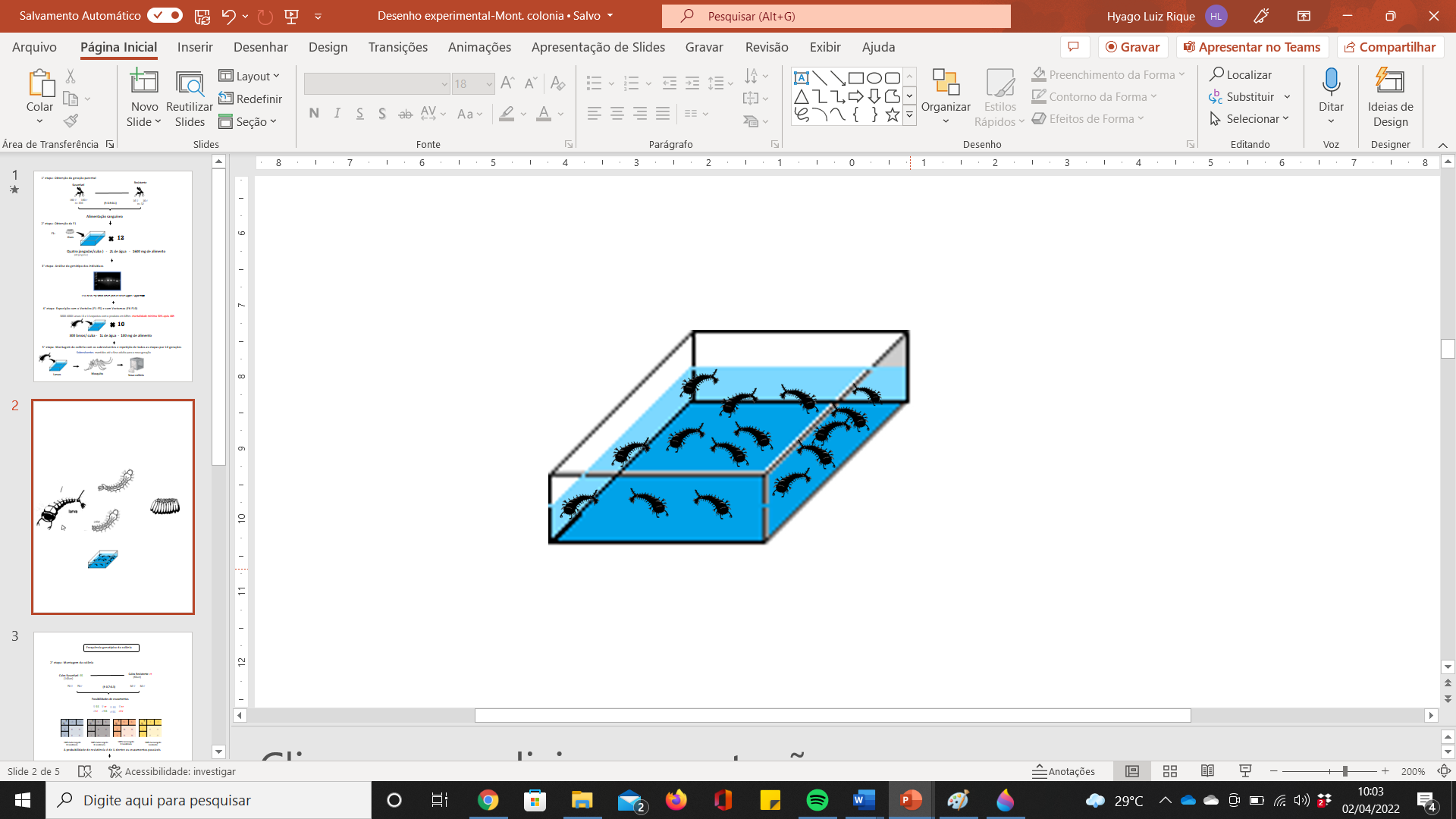


Blood meal


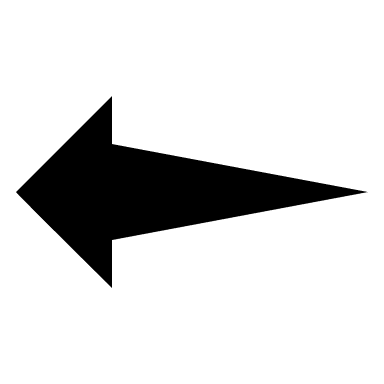


Progeny: larvae rearing


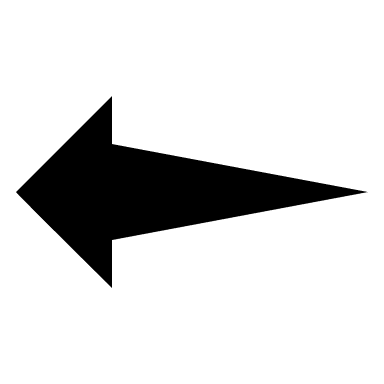


Genotype *cqm1*/*cqm1*_REC_ alleles


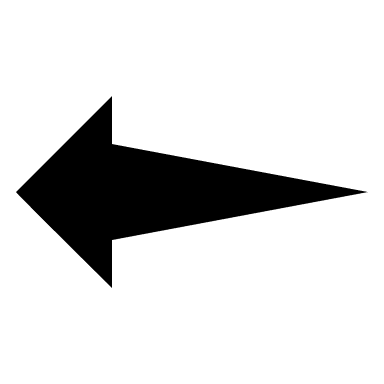

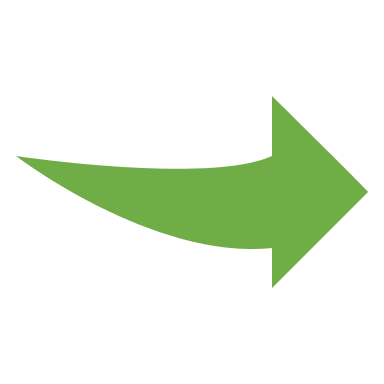


*L. sphaericus or*

*L. sphaericus/Bti*


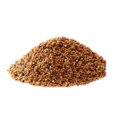

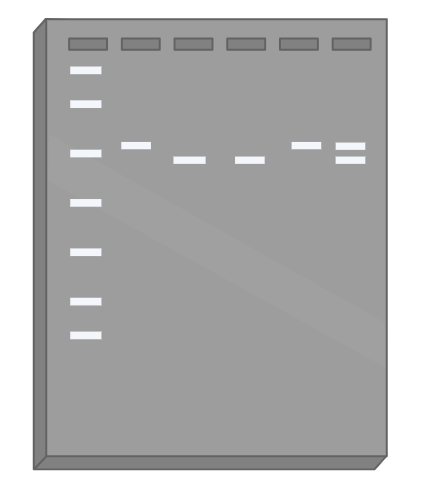

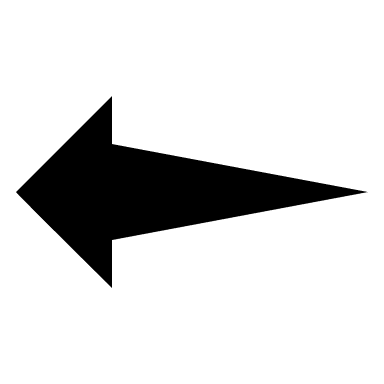


Mortality record

Surviving adults


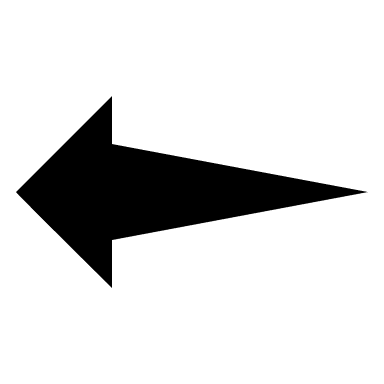

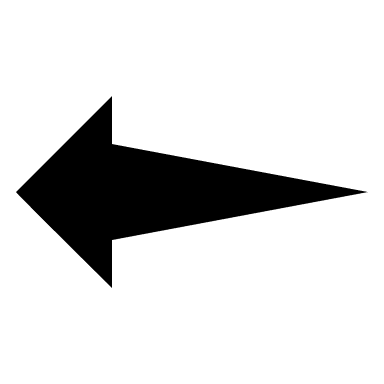

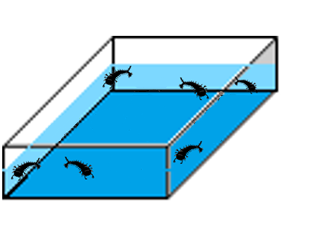


N~ 50

N~ 100

**Additional file 1: Figure S1**. Representation of the procedures for the maintenance of the *Culex quinquefasciatus* SREC strain. The strain was composed of susceptible and Bin-resistant individuals, treated with the *Lysinibacillus sphaericus* or the *L. sphaericus*/*Bacillus thuringiensis* svar. *israelensis* larvicides. The genotypes for *cqm1* and *cqm1*_REC_ alleles were evaluated for larvae before the treatment and for adults surviving to the treatment, across four generations
