# Supplementary material for: Evaluation of a long-lasting microbial larvicide against Culex quinquefasciatus and Aedes aegypti under laboratory and a semi-field trial
Source: Parasit Vectors. 2024 Sep 14;17:391. doi: 10.1186/s13071-024-06465-5 (PMC11401406; doi:10.1186/s13071-024-06465-5)
Supplement: Supplementary file 2 — Additional file 2: Table S1. Dataset of the dose–response bioassays of the Lysinibacillus sphaericus/Bacillus thuringiensis svar. israelensis against mosquito larvae. [file 13071_2024_6465_MOESM2_ESM.docx]

**Additional file 2: Table S1.** Dataset of the dose-response bioassays of the *Lysinibacillus sphaericus*/*Bacillus thuringiensis* svar. *israelensis* against mosquito larvae.

| Colony | No larvae | LC_50_ (CI95%) ^1^ | LC_90_ (CI95%) ^1^ | Slope | *Х*^2^ /df ^2^ |
| --- | --- | --- | --- | --- | --- |
| CqS | 360 | 0.010 (0.008-0.011) | 0.041 (0.030-0.063) | 2.0 | 1.30 |
|  | 420 | 0.004 (0.003-0.005) | 0.015 (0.012-0.020) | 2.3 | 0.23 |
|  | 360 | 0.006 (0.004-0.007) | 0.035 (0.025-0.057) | 1.6 | 0.58 |
| Total | 1140 | 0.006 (0.003-0.011) | 0.030 (0.012-0.063) |  |  |
| CqR | 360 | 0.006 (0.005-0.008) | 0.032 (0.023-0.051) | 1.8 | 1.07 |
|  | 360 | 0.010 (0.007-0.013) | 0.104 (0.059-0.269) | 1.2 | 0.17 |
|  | 300 | 0.013 (0.010-0.017) | 0.091 (0.055-0.228) | 1.5 | 1.14 |
|  | 240 | 0.008 (0.006-0.010) | 0.052 (0.032-0.109) | 1.6 | 0.46 |
| Total | 1260 | 0.009 (0.005-0.017) | 0.069 (0.023-0.269) |  |  |
| Rocke | 360 | 0.036 (0.032-0.040) | 0.093 (0.075-0.131) | 3.1 | 0.50 |
|  | 420 | 0.038 (0.035-0.041) | 0.071 (0.064-0.083) | 4.7 | 0.62 |
|  | 360 | 0.048 (0.043-0.053) | 0.098 (0.085-0.119) | 4.1 | 0.45 |
|  | 360 | 0.046 (0.043-0.050) | 0.082 (0.074-0.097) | 5.0 | 0.50 |
| Total | 1500 | 0.042 (0.032-0.053) | 0.086 (0.064-0.131) |  |  |

The toxicity was evaluated to third instar larvae of *Culex quinquefasciatus* susceptible (CqS), resistant to the Binary toxin (CqR) and *Aedes aegypti* (Rocke). ^1^ Lethal Concentration (mg/L) for 50% to 90% of larvae after 48h of exposure with a 95% confidence interval. ^2^ Heterogeneity data obtained from the ratio between chi-square (*Х*^2^) and degrees of freedom (df).
