# Supplementary material for: Evaluation of a long-lasting microbial larvicide against Culex quinquefasciatus and Aedes aegypti under laboratory and a semi-field trial
Source: Parasit Vectors. 2024 Sep 14;17:391. doi: 10.1186/s13071-024-06465-5 (PMC11401406; doi:10.1186/s13071-024-06465-5)
Supplement: Supplementary file 3 — Additional file 3: Table S2. Dataset of the diagnostic bioassays of the Lysinibacillus sphaericus and/or Bacillus thuringiensis svar. israelensis against mosquito larvae. [file 13071_2024_6465_MOESM3_ESM.docx]

**Additional file 3: Table S2**. Dataset of the diagnostic bioassays of the *Lysinibacillus sphaericus* and/or *Bacillus thuringiensis* svar. *israelensis* against mosquito larvae.

|  |  | Mortality | | | | | | | | | | | | |
| --- | --- | --- | --- | --- | --- | --- | --- | --- | --- | --- | --- | --- | --- | --- |
|  |  |  | | | Replicates | | | | | | | | | |
| Strain  Larvicide  (mg/L) | No.  larvae | No. | Mean  % | | 1 | 2 | 3 | 4 | 5 | 6 | 7 | 8 | 9 | 10 |
| CqS |  |  |  |  | |  |  |  |  |  |  |  |  |  |
| Lsp/Bti |  |  |  |  | |  |  |  |  |  |  |  |  |  |
| 0.005 | 1200 | 74 | 6.2 | 26 | | 13 | 23 | 12 | - | - | - | - | - | - |
| 0.01 | 900 | 638 | 70.9 | 191 | | 226 | 221 | - | - | - | - | - | - | - |
| 0.02 | 900 | 722 | 80.2 | 237 | | 233 | 252 | - | - | - | - | - | - | - |
| 0.06 | 600 | 563 | 93.8 | 276 | | 287 | - | - | - | - | - | - | - | - |
| 0.1 | 900 | 895 | 99.4 | 99 | | 100 | 100 | 97 | 100 | 100 | 99 | 100 | 100 | - |
| Lsp |  |  |  |  | |  |  |  |  |  |  |  |  |  |
| 0.005 | 900 | 759 | 84.3 | 244 | | 267 | 248 | - | - | - | - | - | - | - |
| 0.01 | 1200 | 1009 | 84.1 | 254 | | 265 | 259 | 231 | - | - | - | - | - | - |
| 0.02 | 900 | 842 | 93.6 | 284 | | 275 | 283 | - | - | - | - | - | - | - |
| 0.03 | 600 | 594 | 99.0 | 294 | | 300 | - | - | - | - | - | - | - | - |
| 0.1 | 900 | 900 | 100.0 | 100 | | 100 | 100 | 100 | 100 | 100 | 100 | 100 | 100 | - |
| CqR |  |  |  |  | |  |  |  |  |  |  |  |  |  |
| Lsp/Bti |  |  |  |  | |  |  |  |  |  |  |  |  |  |
| 0.06 | 1800 | 899 | 49.9 | 153 | | 162 | 131 | 166 | 134 | 153 | - | - | - | - |
| 0.1 | 900 | 875 | 97.2 | 94 | | 100 | 94 | 92 | 99 | 100 | 98 | 98 | 100 | - |
| Lsp |  |  |  |  | |  |  |  |  |  |  |  |  |  |
| 0.1 | 900 | 2 | 0.2 | 0 | | 0 | 2 | 0 | 0 | 0 | 0 | 0 | 0 |  |
| Rocke |  |  |  |  | |  |  |  |  |  |  |  |  |  |
| Lsp/Bti |  |  |  |  | |  |  |  |  |  |  |  |  |  |
| 0.1 | 700 | 333 | 47.6 | 42 | | 40 | 59 | 51 | 49 | 44 | 48 | - | - | - |
| 0.2 | 1000 | 964 | 96.4 | 100 | | 83 | 81 | 100 | 100 | 100 | 100 | 100 | 100 | 100 |
| Lsp |  |  |  |  | |  |  |  |  |  |  |  |  |  |
| 0.1 | 1000 | 2 | 0.2 | 0 | | 0 | 0 | 0 | 0 | 0 | 2 | 0 | 0 | 0 |
| 0.2 | 500 | 23 | 4.6 | 0 | | 7 | 5 | 4 | 7 | - | - | - | - | - |

Toxicity assays of *Lysinibacillus sphaericus*/*Bacillus thuringiensis* svar. *israelensis* larvicide (Lsp/Bti) and *L. sphaericus* (Lsp) larvicides against third instar larvae of *Culex quinquefasciatus* susceptible (CqS) and resistant to the Binary toxin (CqR) and *Aedes aegypti* (Rocke). Each concentration was tested using at least three technical replicates, and each replicate had 100 or 300 larvae in trays in 1L of water with food (0.05-0.1 g). The mortality was determined after 48h.
