# Supplementary material for: Evaluation of a long-lasting microbial larvicide against Culex quinquefasciatus and Aedes aegypti under laboratory and a semi-field trial
Source: Parasit Vectors. 2024 Sep 14;17:391. doi: 10.1186/s13071-024-06465-5 (PMC11401406; doi:10.1186/s13071-024-06465-5)
Supplement: Supplementary file 4 — Additional file 4: Table S3. Dataset of the residual activity of Lysinibacillus sphaericus/Bacillus thuringiensis svar. israelensis larvicide toward mosquito larvae. [file 13071_2024_6465_MOESM4_ESM.docx]

**Additional file 4: Table S3**. Dataset of the residual activity of *Lysinibacillus sphaericus*/*Bacillus thuringiensis* svar. *israelensis* larvicide towards mosquito larvae.

|  |  |  |  |  | Mortality | | | | | | | | | | | | | | | | | | | | | | | |
| --- | --- | --- | --- | --- | --- | --- | --- | --- | --- | --- | --- | --- | --- | --- | --- | --- | --- | --- | --- | --- | --- | --- | --- | --- | --- | --- | --- | --- |
| Weeks | ° C ^1^  (in/out) | H%^2^ | pH | T ^3^  (g/100L) | *Cx. quinquefasciatus* (n= 100) | | | | | | | | | | | | | *Ae. aegypti* (n= 50) | | | | | | | | | | |
|  |  |  |  |  | Total | X%^4^ | SD^5^ | R1 | R2 | R3 | R4 | R5 | R6 | R7 | R8 |  | Total | | X% | SD | R1 | R2 | R3 | R4 | R5 | R6 | R7 | R8 |
| 0 | 28.9/28.6 | 57 | 5 | C^6^ | 4 | 1 | 1 | 0 | 2 | 0 | 2 |  |  |  |  |  | 0 | | 0 | 0 | 0 | 0 | 0 | 0 |  |  |  |  |
|  | 29.1/28.5 |  | 5 | 2 | 400 | 100 | 0 | 100 | 100 | 100 | 100 |  |  |  |  |  | 200 | | 100 | 0 | 50 | 50 | 50 | 50 |  |  |  |  |
|  | 29.3/28.1 |  | 5 | 4 | 400 | 100 | 0 | 100 | 100 | 100 | 100 |  |  |  |  |  | 200 | | 100 | 0 | 50 | 50 | 50 | 50 |  |  |  |  |
| 1 | 28.1/27.7 | 54 | 5 | C | 9 | 2 | 2 | 6 | 0 | 2 | 1 |  |  |  |  |  | 1 | | 1 | 0 | 0 | 1 | 0 | 0 |  |  |  |  |
|  | 30.9/28.6 |  | 5 | 2 | 397 | 99 | 1 | 98 | 99 | 100 | 100 |  |  |  |  |  | 200 | | 100 | 0 | 50 | 50 | 50 | 50 |  |  |  |  |
|  | 31.7/28 |  | 5 | 4 | 400 | 100 | 0 | 100 | 100 | 100 | 100 |  |  |  |  |  | 200 | | 100 | 0 | 50 | 50 | 50 | 50 |  |  |  |  |
| 2 | 26.3/27.7 | 56 | 5 | C | 28 | 7 | 2 | 9 | 6 | 5 | 8 |  |  |  |  |  | 12 | | 6 | 1 | 1 | 4 | 3 | 4 |  |  |  |  |
|  | 28.2/27.7 |  | 5 | 2 | 391 | 98 | 4 | 91 | 100 | 100 | 100 |  |  |  |  |  | 200 | | 100 | 0 | 50 | 50 | 50 | 50 |  |  |  |  |
|  | 28.6/27.6 |  | 5 | 4 | 400 | 100 | 0 | 100 | 100 | 100 | 100 |  |  |  |  |  | 200 | | 100 | 0 | 50 | 50 | 50 | 50 |  |  |  |  |
| 3 | 27.3/27.6 | 77 | 5 | C | 69 | 17 | 28 | 3 | 0 | 66 | 0 |  |  |  |  |  | 20 | | 10 | 6 | 6 | 0 | 14 | 0 |  |  |  |  |
|  | 27.7/27.2 |  | 5 | 2 | 365 | 91 | 10 | 74 | 94 | 100 | 97 |  |  |  |  |  | 187 | | 94 | 4 | 40 | 47 | 50 | 50 |  |  |  |  |
|  | 27.7/27.3 |  | 5 | 4 | 398 | 100 | 1 | 98 | 100 | 100 | 100 |  |  |  |  |  | 200 | | 100 | 0 | 50 | 50 | 50 | 50 |  |  |  |  |
| 4 | 31.3/28 | 52 | 5 | C | 181 | 45 | 26 | 54 | 0 | 60 | 67 |  |  |  |  |  | 31 | | 16 | 6 | 4 | 0 | 10 | 17 |  |  |  |  |
|  | 31.3/28.5 |  | 5 | 2 | 364 | 91 | 10 | 74 | 91 | 100 | 99 |  |  |  |  |  | 188 | | 94 | 3 | 43 | 45 | 50 | 50 |  |  |  |  |
|  | 31.5/29 |  | 5 | 4 | 397 | 99 | 1 | 98 | 100 | 99 | 100 |  |  |  |  |  | 200 | | 100 | 0 | 50 | 50 | 50 | 50 |  |  |  |  |
| 5 | 29.3/27 | 59 | 5 | C | 141 | 35 | 35 | 71 | 70 | - | - | 0 | 0 |  |  |  | 26 | | 13 | 7 | - | - | 9 | 17 | 0 | 0 |  |  |
|  | 29.9/27 |  | 5 | 2 | 363 | 91 | 9 | 75 | 93 | 99 | 96 |  |  |  |  |  | 167 | | 84 | 10 | 25 | 45 | 50 | 47 |  |  |  |  |
|  | 30.1/24 |  | 5 | 4 | 400 | 100 | 0 | 100 | 100 | 100 | 100 |  |  |  |  |  | 197 | | 99 | 1 | 48 | 49 | 50 | 50 |  |  |  |  |
| 6 | 27/26.9 | 71 | 5 | C | 7 | 2 | 1 | - | - | - | - | 2 | 2 | 3 | 0 |  | 6 | | 3 | 2 | - | - | - | - | 0 | 3 | 3 | 0 |
|  | 27/27 |  | 5 | 2 | 331 | 83 | 11 | 73 | 71 | 92 | 95 |  |  |  |  |  | 178 | | 89 | 7 | 32 | 46 | 50 | 50 |  |  |  |  |
|  | 27/27.1 |  | 5 | 4 | 394 | 99 | 1 | 98 | 100 | 97 | 99 |  |  |  |  |  | 200 | | 100 | 0 | 50 | 50 | 50 | 50 |  |  |  |  |
| 7 | 28.2/27.5 | 69 | 5 | C | 145 | 36 | 37 | - | - | - | - | 0 | 63 | 82 | 0 |  | 34 | | 17 | 11 | - | - | - | - | 0 | 7 | 27 | 0 |
|  | 28.4/27.6 |  | 5 | 2 | 327 | 82 | 13 | 63 | 77 | 91 | 96 |  |  |  |  |  | 131 | | 66 | 17 | 7 | 27 | 48 | 49 |  |  |  |  |
|  | 29.4/28 |  | 5 | 4 | 387 | 97 | 5 | 88 | 100 | 100 | 99 |  |  |  |  |  | 192 | | 96 | 1 | 47 | 48 | 50 | 47 |  |  |  |  |
| 8 | 28.3/29.3 | 70 | 5 | C | 0 | 0 | 0 | - | - | - | - | 0 | 0 | 0 | 0 |  | 0 | | 0 | 0 | - | - | - | - | 0 | 0 | 0 | 0 |
|  | 30.9/29 |  | 5 | 2 | 287 | 72 | 6 | 64 | 69 | 75 | 79 |  |  |  |  |  | 112 | | 56 | 13 | 8 | 25 | 37 | 42 |  |  |  |  |
|  | 32/29 |  | 5 | 4 | 357 | 89 | 10 | 74 | 94 | 89 | 100 |  |  |  |  |  | 165 | | 83 | 13 | 19 | 49 | 47 | 50 |  |  |  |  |
| 9 | 28.9/28.7 | 67 | 5 | C | 3 | 1 | 1 | - | - | - | - | 0 | 3 | 0 | 0 |  | 0 | | 0 | 0 | - | - | - | - | 0 | 0 | 0 | 0 |
|  | 28.5/28.3 |  | 5 | 2 | 302 | 76 | 4 | 70 | 74 | 78 | 80 |  |  |  |  |  | 118 | | 59 | 13 | 17 | 17 | 36 | 48 |  |  |  |  |
|  | 29/28.7 |  | 5 | 4 | 342 | 86 | 11 | 67 | 90 | 88 | 97 |  |  |  |  |  | 155 | | 78 | 18 | 8 | 50 | 47 | 50 |  |  |  |  |
| **10** | 28.2/28.9 | 71 | 5 | C | 55 | 14 | 22 | - | - | - | - | 0 | 3 | 51 | 1 |  | 17 | | 9 | 6 | - | - | - | - | 1 | 1 | 15 | 0 |
|  | 29.6/28.7 |  | 5 | 2 | 279 | 70 | 5 | 61 | 71 | 74 | 73 |  |  |  |  |  | 115 | | 58 | 12 | 27 | 15 | 26 | 47 |  |  |  |  |
|  | 29.8/28.8 |  | 5 | 4 | 310 | 78 | 7 | 66 | 81 | 84 | 79 |  |  |  |  |  | 134 | | 67 | 17 | 5 | 42 | 49 | 38 |  |  |  |  |
| **11** | 31/29.5 | 69 | 5 | C | 111 | 28 | 34 | - | - | - | - | 8 | 17 | 85 | 1 |  | 18 | | 9 | 6 | - | - | - | - | 4 | 0 | 14 | 0 |
|  | 31.8/28.3 |  | 5 | 2 | 301 | 75 | 1 | 76 | 75 | 77 | 73 |  |  |  |  |  | 110 | | 55 | 12 | 18 | 16 | 31 | 45 |  |  |  |  |
|  | 31.8/28.3 |  | 5 | 4 | 309 | 77 | 7 | 72 | 77 | 71 | 89 |  |  |  |  |  | 143 | | 72 | 17 | 6 | 48 | 41 | 48 |  |  |  |  |
| **12** | 27.6/28.7 | 75 | 5 | C | 74 | 25 | 35 | - | - | - | - | 0 | ND^6^ | 74 | 0 |  | 19 | | 6 | 9 | - | - | - | - | 0 | ND^6^ | 19 | 0 |
|  | 26.7/28.6 |  | 5 | 2 | 289 | 72 | 4 | 66 | 73 | 76 | 74 |  |  |  |  |  | 107 | | 54 | 11 | 18 | 14 | 34 | 41 |  |  |  |  |
|  | 29.2/28 |  | 5 | 4 | 319 | 80 | 8 | 70 | 83 | 75 | 91 |  |  |  |  |  | 135 | | 68 | 10 | 18 | 34 | 36 | 47 |  |  |  |  |

The activity in this simulated field trial was evaluated against third instar larvae of *Culex quinquefasciatus* (susceptible-CqS, Bin-resistant CqR) and *Aedes aegypti* (Rocke). A single treatment (T) in 100L containers with 150 larvae (CqS= 70, CqR= 30, Rocke= 50) was done, a new set of larvae was introduced every week and the average mortality in the four replicates (R) of the treated and untreated control groups (C) was recorded every after recolonization weekly. When the mortality exceeded 20% in the untreated control containers they were replaced^1^ Temperature in the water and outside the water. ^2^ Humidity. ^3^ Treatment. ^4^ Average of replicates. ^5^ Standard deviation of replicates. ^6^ Not determined, excluded due to water turbidity.
