# Supplementary material for: Evaluation of a long-lasting microbial larvicide against Culex quinquefasciatus and Aedes aegypti under laboratory and a semi-field trial
Source: Parasit Vectors. 2024 Sep 14;17:391. doi: 10.1186/s13071-024-06465-5 (PMC11401406; doi:10.1186/s13071-024-06465-5)
Supplement: Supplementary file 5 — Additional file 5: Figure S2. Dataset of the residual activity of Lysinibacillus sphaericus/Bti to control mosquito larvae. [file 13071_2024_6465_MOESM5_ESM.docx]

**Additional file 5: Figure S2**. Dataset of the residual activity of *Lysinibacillus sphaericus*/Bti to control mosquito larvae. (A) Treatment at 2 g/100L. (B) Treatment at 4 g/100L. Groups of *Culex quinquefasciatus* larvae (70 susceptible larvae and 30 Bin-resistant larvae/container) and *Aedes aegypti* mosquito larvae (50 larvae/container), in quadruplicate, kept in semi-field conditions for twelve weeks. Each bar from a given color represents a replicate of each treatment tested: R1, R2, R3 and R4. The line indicates 80% mortality.

**B**

**A**
