# Supplementary material for: Evaluation of a long-lasting microbial larvicide against Culex quinquefasciatus and Aedes aegypti under laboratory and a semi-field trial
Source: Parasit Vectors. 2024 Sep 14;17:391. doi: 10.1186/s13071-024-06465-5 (PMC11401406; doi:10.1186/s13071-024-06465-5)
Supplement: Supplementary file 6 — Additional file 6: Figure S3. Nucleotide sequences of amplicons of the Culex quinquefasciatus cqm1 gene from SREC individuals. [file 13071_2024_6465_MOESM6_ESM.docx]

1210 1220 1230 1240 1250 1260 1270 1280 1290 1300

....|....|....|....|....|....|....|....|....|....|....|....|....|....|....|....|....|....|....|....|

**Cqm1(S).ref**  **GCTCAGCAGTTCGACAACCGAGATCCGAACCGGACGCCGATGCAGTGGGACTCGTCGACCAGTGCCGGGTTCAGTACCAACACCAACACCTGGCTCCGAG**

**S1.seq**  **GCTCAGCAGTTCGACAACCGAGATCCGAACCGGACGCCGATGCAGTGGGACTCGTCGACCAGTGCCGGGTTCAGTACCAACACCAACCCTG---------**

**S2.seq**  **GCTCAGCAGTTCGACAACCGAGATCCGAACCGGACGCCGATGCAGTGGGACTCGTCGACCAGTGCCGGGTTCAGTACCAACACCAACCCTGGCTCCGAGT**

**S3.seq**  **GCTCAGCAGTTCGACAACCGAGATCCGAACCGGACGCCGATGCAGTGGGACTCGTCGACCAGTGCCGGGTTCAGTACCAACACCAACACCTGGCTCCGAG**

**S4.seq**  **GCTCAGCAGTTCGACAACCGAGATCCGAACCGGACGCCGATGCAGTGGGACTCGTCGACCAGTGCCGGGTTCAGTACCAACACCAACC------------**

**S5.seq**  **GCTCAGCAGTTCGACAACCGAGATCCGAACCGGACGCCGATGCAGTGGGACTCGTCGACCAGTGCCGGGTTCAGTACCAACACCAACACCTGGCTCCGAG**

**S6.seq**  **GCTCAGCAGTTCGACAACCGAGATCCGAACCGGACGCCGATGCAGTGGGACTCGTCGACCAGTGCCGGGTTCAGTACCAACACCAACACCTGGCTCCG--**

**S7.seq**  **GCTCAGCAGTTCGACAACCGAGATCCGAACCGGACGCCGATGCAGTGGGACTCGTCGACCAGTGCCGGGTTCAGTACCAACACCAACACCTGGCTCCGAG**

**S8.seq**  **GCTCAGCAGTTCGACAACCGAGATCCGAACCGGACGCCGATGCAGTGGGACTCGTCGACCAGTGCCGGGTTCAGTACCAACACCAACTCCTGGCTCCGAG**

**S9.seq**  **GCTCAGCAGTTCGACAACCGAGATCCGAACCGGACGCCGATGCAGTGGGACTCGTCGACCAGTGCCGGGTTCAGTACCAACACCAACACCTGGCTCCGAG**

**S10.seq**  **GCTCAGCAGTTCGACAACCGAGATCCGAACCGGACGCCGATGCAGTGGGACTCGTCGACCAGTGCCGGGTTCAGTACCAACACCAACACCTGGCTCCGAG**

**Cqm1(REC).ref** **GCTCAGCAGTTCGACAACCGAGACT-------------------GTGGGACTCGTCGACCAGTGCCGGGTTCAGTACCAACACCAACACCTGGCTCCGAG**

**R1.seq**  **GCTCAGCAGTTCGACAACCGAGACT-------------------GTGGGACTCGTCGACCAGTGCCGGGTTCAGTACCAACACCAACACCTGGCTCCGAG**

**R2.seq**  **GCTCAGCAGTTCGACAACCGAGACT-------------------GTGGGACTCGTCGACCAGTGCCGGGTTCAGTACCAACACCAACACCTGGCTCCGAG**

**R3.seq**  **GCTCAGCAGTT-GACAACCGAGACT-------------------GTGGGACT-GTCGACCAGTGCCGGGTTCAGTACCAACACCAACACCTGGCTCCGAG**

**R4.seq**  **GCTCAGCAGTT-GACAACCGAGACT-------------------GTGGGACT-GTCGACCAGTGCCGGGTTCAGTACCAACACCAACACCTGGCTCCGAG**

**R5.seq**  **G-TCAGCAGTT-GACAAC-GAGA-T-------------------GTGGGACT-GT-GACCAGTGCCGGGTTCAGTACCAACACCAACACCTGGCTCCGAG**

**R6.seq**  **G-TCAGCAGTT-GACAACCGAGACT-------------------GTGGGACT-GTCGACCAGTGCCGGGTTCAGTACC--CACCAACACC--GGTCCGAG**

**R7.seq**  **G-TCAGCAGTT-GACAACCGAGAT--------------------GTGGGACT-GTCGACCAGTGCCGGGTTCAG-ACCAACACCAACACC-GGCTCCGAG**

**Additional file 6: Figure S3**. Nucleotide sequences of amplicons of the *Culex quinquefasciatus cqm1* gene from SREC individuals. Reference sequences (ref) of the *cqm1* 208 nt amplicon (S) and the *cqm1_REC_* 189 nt amplicon (Gene Bank accession number DQ333335). Sequences of the SREC individuals that produced 189 nt (R1-R7) and 208 nt (S1-S10) amplicons. Identical nucleotides (black), 19 nt deletion (red box), and gaps (-) are indicated.
