# Supplementary material for: Evaluation of a long-lasting microbial larvicide against Culex quinquefasciatus and Aedes aegypti under laboratory and a semi-field trial
Source: Parasit Vectors. 2024 Sep 14;17:391. doi: 10.1186/s13071-024-06465-5 (PMC11401406; doi:10.1186/s13071-024-06465-5)
Supplement: Supplementary file 7 — Additional file 7: Table S4. Mortality of Culex quinquefasciatus larvae from the SREC strain from four generations (F). [file 13071_2024_6465_MOESM7_ESM.docx]

**Additional file 7: Table S****4.** Mortality of *Culex quinquefasciatus* larvae from the SREC strain from four generations (F).

|  | Treatment | |  | Mortality | |
| --- | --- | --- | --- | --- | --- |
| Samples | Larvicide (mg/L) | No. larvae treated |  | 24h | Total |
| F_1_ | Lsp (0.005) | 3600 |  | 59.6 | 86.2 |
| F_2_ | Lsp/Bti (0.01) | 3000 |  | 12.5 | 47.1 |
| F_3_ | Lsp/Bti (0.01) | 3000 |  | 5.2 | 26.7 |
| F_4_ | Lsp (0.1) | 1200 |  | 53.3 | NA^3^ |
|  | Lsp/Bti (0.1) | 1200 |  | 95 | NA |
| S | Lsp (0.1) | 900 |  | 99.8 | NA |
|  | Lsp/Bti (0.1) | 900 |  | 99.9 | NA |

Samples were treated with *Lysinibacillus sphaericus* (Lsp-VectoLex WG®) or *Lysinibacillus sphaericus*/*Bacillus thuringiensis* svar. *israelensis* (Lsp/Bti-VectoMax FG™) larvicides. The mortality was recorded 48h after the treatment and until adult emergence (total). Positive control samples to show the toxicity of both larvicides are larvae from the susceptible reference strain (S). NA. Not applicable.
