# Supplementary material for: Evaluation of a long-lasting microbial larvicide against Culex quinquefasciatus and Aedes aegypti under laboratory and a semi-field trial
Source: Parasit Vectors. 2024 Sep 14;17:391. doi: 10.1186/s13071-024-06465-5 (PMC11401406; doi:10.1186/s13071-024-06465-5)
Supplement: Supplementary file 8 — Additional file 8: Table S5. Frequency of the genotypes for the cqm1 and cqm1REC alleles in Culex quinquefasciatus SREC2 strain. [file 13071_2024_6465_MOESM8_ESM.docx]

**Additional file 8: Table S5**. Frequency of the genotypes for the *cqm1* and *cqm1_REC_* alleles in *Culex quinquefasciatus* SREC2 strain.

|  |  | Frequency in larvae | | | | | | |  | Frequency in adults | | | | | | |
| --- | --- | --- | --- | --- | --- | --- | --- | --- | --- | --- | --- | --- | --- | --- | --- | --- |
|  |  |  | Genotype | | |  | Allele | |  |  | Genotype | | |  | Allele | |
| Samples |  | No | SS | SR | RR |  | S | R |  | No | SS | SR | RR |  | S | R |
| P |  | NA | NA | NA | NA |  | NA | NA |  | 30 | 0.70 | 0.00 | 0.30 |  | 0.70 | 0.30 |
| F_1_ |  | 100 | 0.61 | 0.30 | 0.09 |  | 0.76 | 0.24 |  | 50 | 0.08 | 0.08 | 0.84 |  | 0.12 | 0.88 |
| F_2_ |  | 50 | 0.00 | 0.02 | 0.98 |  | 0.01 | 0.99 |  | 51 | 0.00 | 0.00 | 1.00 |  | 0.00 | 1.00 |
| F3 |  | ND^3^ | ND | ND | ND |  | ND | ND |  | NA | NA | NA | NA | NA | NA | NA |

Note: The genotypes are susceptible homozygous (SS), susceptible heterozygous (SR), and resistant homozygous (RR). Parental generation (P) was set with SS and RR adults. At each generation (F) larvae were treated and the mortality was recorded (Table 3). The genotypes of larvae were determined before treatment and the genotypes surviving adults were assessed after treatment. NA: not applicable.
