# Supplementary material for: Evaluation of a long-lasting microbial larvicide against Culex quinquefasciatus and Aedes aegypti under laboratory and a semi-field trial
Source: Parasit Vectors. 2024 Sep 14;17:391. doi: 10.1186/s13071-024-06465-5 (PMC11401406; doi:10.1186/s13071-024-06465-5)
Supplement: Supplementary file 10 — Additional file 10: Figure S4. Genotypes for the cqm1 and cqm1REC alleles in individuals from the Culex quinquefasciatus SREC2 strain. [file 13071_2024_6465_MOESM10_ESM.docx]

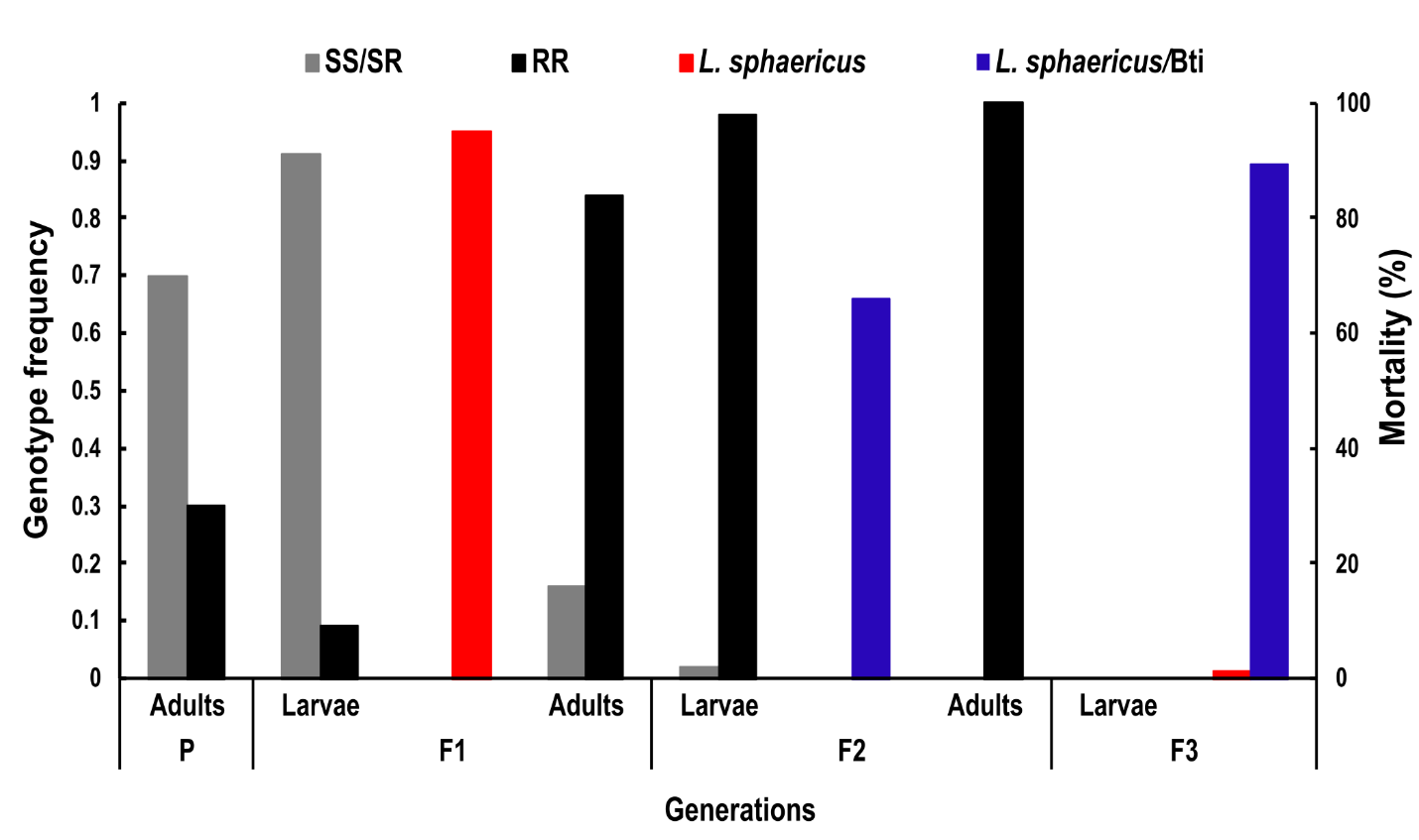


**Additional file 10: Figure S4.** Genotypes for the *cqm1* and *cqm1_REC_* alleles in individuals from the *Culex quinquefasciatus* SREC2 strain. The parental generation (P) was established with homozygous susceptible and homozygous resistant adults. The genotypes of larvae samples (n~ 100) were determined at each generation (F), before treatment. Larvae from each generation were treated with *Lysinibacillus sphaericus-*VectoLex WG® (F_1_) or *L. sphaericus*/*Bacillus thuringiensis* svar. *israelensis-*VectoMax FG™ (F_2_-F_4_). The mortality was recorded and the genotypes of a sample of surviving adults (n~ 50) was assessed. Full dataset is available in Additional file 8 Table S6 and Additional file 9: Table S7.
